# LncRNA KLF3-AS1 in human mesenchymal stem cell-derived exosomes ameliorates pyroptosis of cardiomyocytes and myocardial infarction through miR-138-5p/Sirt1 axis

**DOI:** 10.1186/s13287-019-1522-4

**Published:** 2019-12-17

**Authors:** Qing Mao, Xiu-Lin Liang, Chuan-Long Zhang, Yi-Heng Pang, Yong-Xiang Lu

**Affiliations:** 1 Department of Cardiology, Nanjing Lishui People’s Hospital, Zhongda Hospital Lishui Branch, Southeast University, No. 86 of Chongwen Road, Lishui District, Nanjing, 211200 Jiangsu People’s Republic of China; 2 Department of Neurology, The Second Affiliated Hospital of Guangxi Medical University, Nanning, 530007 Guangxi People’s Republic of China; 3 Department of Cardiology, The Second Affiliated Hospital of Guangxi Medical University, Nanning, 530007 Guangxi People’s Republic of China

**Keywords:** Exosome, Pyroptosis, Myocardial infarction, Human mesenchymal stem cells, KLF3-AS1, miR-138-5p, Sirt1, lncRNA

## Abstract

**Aim:**

Myocardial infarction (MI) is a severe disease with increased mortality and disability rates, posing heavy economic burden for society. Exosomes were uncovered to mediate intercellular communication after MI. This study aims to explore the effect and mechanism of lncRNA KLF3-AS1 in exosomes secreted by human mesenchymal stem cells (hMSCs) on pyroptosis of cardiomyocytes and MI.

**Methods:**

Exosomes from hMSCs were isolated and identified. Exosomes from hMSCs with transfection of KLF3-AS1 for overexpression were injected into MI rat model or incubated with hypoxia cardiomyocytes. Effect of KLF3-AS1 on MI area, cell viability, apoptosis, and pyroptosis was determined. The relationship among miR-138-5p, KLF3-AS1, and Sirt1 was verified by dual-luciferase reporter assay. Normal cardiomyocytes were transfected with miR-138-5p inhibitor or sh-Sirt1 to clarify whether alteration of miR-138-5p or sh-Sirt1 can regulate the effect of KLF3-AS1 on cardiomyocytes.

**Results:**

Exosomes from hMSCs were successfully extracted. Transfection of KLF3-AS1 exosome in rats and incubation with KLF3-AS1 exosome in hypoxia cardiomyocytes both verified that overexpression of KLF3-AS1 in exosomes leads to reduced MI area, decreased cell apoptosis and pyroptosis, and attenuated MI progression. KLF3-AS1 can sponge miR-138-5p to regulate Sirt1 expression. miR-138-5p inhibitor transfection and KLF3-AS1 exosome incubation contribute to attenuated pyroptosis and MI both in vivo and in vitro, while transfection of sh-Sirt1 could reverse the protective effect of exosomal KLF3-AS1 on hypoxia cardiomyocytes.

**Conclusion:**

LncRNA KLF3-AS1 in exosomes secreted from hMSCs by acting as a ceRNA to sponge miR-138-5p can regulate Sirt1 so as to inhibit cell pyroptosis and attenuate MI progression.

## Introduction

Myocardial infarction (MI) is a severe coronary artery-related disease, mainly resulted from disruption of coronary atherothrombosis or imbalance of myocardial oxygen supply-demand [1–3]. Upon disruption of atherothrombosis, the released plaque could accumulate platelet, leading to coronary artery occlusion and consequently resulting in myocardial ischemia and necrosis [4]. Various cell death mechanisms, including apoptosis, pyroptosis, and necrocytosis, can be activated in response to myocardial ischemia when nutrients and oxygen are deprived [5]. Pyroptosis is initially identified as a host defensive response to remove various bacterial and viral infections while pathogens have also developed mechanisms to inhibit cell pyroptosis so as to enhance their ability to persist and cause disease [6]. Pyroptosis is a programmed cell death accompanied by inflammasome-induced inflammatory caspases in the caspase-1 family [7, 8]. GSDMD, an effector of pyroptosis, can be cleaved by caspase-1 which allows the releasing of N-terminal pore-forming domain to insert into the plasma membrane [9, 10]. Gasdermin D pores mediate osmotic cell swelling, rupture of the plasma membrane, and release of intracellular contents including the enzyme lactate dehydrogenase. Therefore, pyroptosis shares the same characteristics with cell apoptosis but featured with cell swelling and plasma membrane disruption [11]. Pyroptosis is involved in the damage of cardiomyocytes, and pyroptosis pathway is a potential therapeutic target for cardiovascular diseases [12]. Prevention or attenuation of cardiomyocyte apoptosis or pyroptosis is necessary to ensure the normal cardiac contraction function after MI [13]. However, the molecular mechanisms underlying pyroptosis in cardiomyocytes are still obscure.

Long non-coding RNAs (lncRNAs) are found to regulate gene expressions by interacting with microRNAs (miRNAs), mRNAs, or proteins [14]. Besides, lncRNAs are reported to play certain roles in mediating cell proliferation, migration, inflammation, apoptosis, and autophagy [15]. Exosomes are mainly responsible for intercellular communication through transferring miRNAs, lncRNAs, and proteins [16–18]. Considering the ability of extracellular vesicles in conveying information, intrigued attention has been paid on the implication and function of miRNAs and lncRNAs in exosomes [19]. Exosomes are membranous vesicles containing various biomolecules including lncRNAs which are involved in cellular communication and secreted from many cells including cancer cells. For instance, exosomes and exosomal lncRNA HIF1A-AS1 were found to be upregulated in patients with atherosclerosis [20]. Mesenchymal stem cells (MSCs) refer to a group of multipotent precursor cells with the capacity to differentiate into various cell types [21]. KLF3-AS1, which localizes at chromosome 4p14 according to the exocarta database, is proved to associate with only osteoarthritis [21]. KLF3-AS1 in exosomes derived from human mesenchymal stem cells (hMSCs) is involved in the induction of chondrocyte proliferation and inhibition of apoptosis through miR-206/GIT1 axis [22]. Nevertheless, evidences regarding the implication of exosomal KLF3-AS1 derived from hMSCs in pyroptosis of cardiomyocytes and MI are lacking.

miRNAs are reported to mediate cell death and proliferation of cardiomyocytes, which play a decisive role in myocardial infarction [13, 23]. Accumulating evidence supported lncRNAs act as competing endogenous RNAs (ceRNAs) in MI to regulate cell biological activities [24]. Online software predicted the binding relationship between KLF3-AS1 and miR-138-5p. Therefore, we hypothesized that exosomal KLF3-AS1 derived from hMSCs by acting as a ceRNA for miR-138-5p may implicate in cardiomyocyte pyroptosis and MI. In this study, we further investigated the potential of exosomal KLF3-AS1 derived from hMSCs in attenuating cardiomyocyte pyroptosis and MI.

## Materials and methods

### Cell culture

hMSCs and rat cardiomyocytes H9c2 were purchased from American Type Culture Collection, ATCC, USA. Those two cells were cultured in DMEM culture medium (Gibco, USA) containing 10% fetal calf serum (FCS) and 1% penicillin/streptomycin at 37 °C in a humidified incubator in which the concentration of CO_2_ was 5%.

### Identification of hMSCs

The hMSCs passaged for four times were counted for cell numbers and adjusted for cell concentration for preparation of 1 × 10^6^/ml cell suspension. Cell morphologies were observed under a microscope. Cell suspensions were inoculated at 6-well plates with 2 ml for each well. In total, 24 wells were inoculated for incubation at 37 °C with 5% CO_2_. The culture medium was replaced every 48 h. Cells were digested by pancreatin at an interval of 24 h. Cells from three duplicate wells were counted for continuously 8 days to calculate the average cell numbers and to draw cell growth curve. After that, 100 μl of cell suspension was added into an Eppendorf tube, in which each 10 μl of CD29-PE, CD44-PE, CD106-FITC, CD34-FITC, or CD45-FITC (Invitrogen, USA) was added and mixed for incubation at room temperature for 30 min. Another Eppendorf tube with cell suspension was set as blank control. PBS washing was performed before the Eppendorf tube was centrifuged at 1000*g* for 5 min. Sediment was re-suspended using PBS. The expressions of exosomal biomarkers, CD29, CD44, CD106, CD34, and CD45 were determined using flow cytometry (FCM).

### Extraction and identification of hMSC-derived exosomes

Exosomes derived from hMSCs were isolated based on the protocol indicated on the exosome isolation kit (Invitrogen, USA). The collected exosome suspensions were diluted with 10 μl of PBS and added on copper grid for reaction at room temperature for 1 min. The exosomes were observed and photographed under a transmission electron microscope (TEM) (Philips, The Netherlands) after negative staining with 3% (w/v) sodium phosphotungstate solution and dd H_2_O wash. About 20 exosomes were randomly selected and subjected to diameter measurement. The expression levels of exosome-specific biomarkers, TSG101 and CD63, were detected by FCM and Western blot. Fluorescence gate setting for FCM with the application of FITC labeled TSG101 or CD63 antibody. After incubation in RNase or RNase + Triton X-100-treated culture medium, the exosomes were subjected to detection of KLF3-AS1 to identify whether KLF3-AS1 is membranous. The expression of KLF3-AS1 in conditioned culture medium was detected at room temperature at 0 h, 4 h, 8 h, and 24 h respectively.

### Exosome labeling

Exosome suspension (100 μl) was mixed with 1 ml of dilution C diluted PKH67 (Sigma) for incubation at room temperature for 4 min. The staining was terminated by adding 1 ml of 0.5% BSA, and the exosomes were re-extracted using extraction kit. The observation under a fluorescence microscope showed that the exosomes were stained by PKH67 (green). H9c2 cells or myocardial tissues were stained with DAPI (Sigma) into blue. PKH67 labeled exosomes were incubated with H9c2 cells for 12 h before locating the exosomes. MI rats were injected with PKH67 labeled exosomes through tail intravenous injection, and then, the exosomes in myocardial tissues were located. H9c2 cells without any treatment were considered as negative control group, while PKH67-labeled H9c2 cells were used as positive control group. PKH67-labeled exosomes incubated with H9c2 cells for respectively 6 h, 12 h, and 24 h were correspondingly grouped according to incubation time.

### Establishment of hypoxia models

In vitro hypoxia cell models were established to simulate myocardial ischemia and infarction in vivo. The hypoxic solution was prepared according Koyama et al. [25]. Cardiomyocytes were incubated with hypoxic solution. The hypoxia models were prepared in a tri-gas incubator (Thermo, USA) with 95% N_2_ + 5% CO_2_ at 37 °C for 6 h.

### Quantitative reverse transcription polymerase chain reaction

Total RNA was extracted using Trizol reagent (Invitrogen) and subjected to purity and concentration measurement using Nanodrop2000 (Thermo, USA). Sequence of target genes was obtained from the NCBI database, and primer5 software was used to design corresponding primers. The obtained RNA was reverse-transcribed into cDNA using PrimeScript™ RT reagent kit (Takara). The PCR amplification was performed in a LightCycler 480 (Roche, Switzerland) based on 40 cycles of 94 °C for 5 min, 94 °C for 5 s, and 60 °C for 35 s. β-actin or U6 is used as internal control. The primer sequences are listed in Table 1. The relative expression levels of RNA were analyzed using 2^−ΔΔCt^ method.

**Table 1:** Primer sequences used for reverse transcription-polymerase chain reaction

Name of primers	Sequences
miR-138-5p-F	GGCCGGACTAAGTGTTGT
miR-138-5p-R	GCAGGGTCCGAGGTATTC
U6-F	CTCGCTTCGGCAGCACATATACT
U6-R	ACGCTTCACGAATTTGCGTGTC
KLF3-AS1-F	CTGTAGGCGCGCTCTTTCTTT
KLF3-AS1-R	TCCGACCAAAGTTTGCCAAG
β-actin-F	TGAGCTGCGTTTTACACCCT
β-actin-R	GTTTGCTCCAACCAACTGCT
Sirt1-F	GTGCAGGTAGTTCCTCGGTG
Sirt1-R	CACAACTCACAGCATGCACAA
caspase-1-F	TAGCTCATTCATCCGTCGGC
caspase-1-R	CACGGTCCCAACAACCGATA
ASC-F	TGGAGGACCTGGAGCGGAGC
ASC-R	CAAGCTGGCTTTTCGTATATTGT
GSDMD-F	GGCAACTTCCAAGTCTCCGA
GSDMD-R	AGTCACACGCAGCATACACA
NLRP3-F	CGGAGAGGTCGTTGGTTCAA
NLRP3-R	TGGCTTTGTGTGAAAAATCCATA

*F* forward, *R* reverse

### Western blot

Suspensions of hMSC-secreted exosomes were lysed in RIPA lysis buffer (Beyotime), and cell lysates were centrifuged at 10,000 *g* for 20 min. The supernatant was then collected for storage at − 80 °C. Suspensions of hMSC-secreted exosomes (16 μl) were loaded with 4 μl of loading buffer (5×, beyotime) at 100 °C water bath for 5 min, followed by centrifugation at 10,000×*g* 5 min at room temperature. Current for electrophoresis was 80 V for 30 min and then 120 V for 90 min. The membranes were transferred at 200 mA for 2 h. After membranes were washed in TBST, 5% fat-free milk was added for blocking for 1 h. The blot was probed with TBST-diluted primary antibodies of rabbit anti-rat β-actin (4970S, Cell Signaling), TSG101 (ab30871, Abcam), CD63 (ab217345, Abcam), Sirt1 (9475T, Cell Signaling), caspase-1 (24232S, Cell Signaling), cleaved-caspase-1 (89332S, Cell Signaling), GSDMD (93709S, Cell Signaling), cleaved-GSDMD (36425S, Cell Signaling), ASC (67824T, Cell Signaling), and NLRP3 (13158S, Cell Signaling) in a shaking bed for incubation for 1 h and subsequently probed with horse radish peroxidase-labeled goat anti-rabbit IgG secondary antibody (1:5000, Beijing ComWin Biotech Co., Ltd., Beijing, China) at room temperature for 1 h. After TBST wash for 3 × 10 min, the secondary antibody signals were detected.

### Cell transfection

KLF3-AS1 overexpression, sh-Sirt1, miR-138-5p inhibitor, miR-138-5p mimic vectors, and corresponding negative controls were synthesized by GenePharma (Shanghai, China). Cells (3 × 10^5^) were seeded into 6-well plates for cell transfection upon cell fusion of 40 ~ 60%. The culture medium was refreshed, and cell transfection buffer was prepared. Tube A was made of 5 μl/well of lipofectamine 2000 (Thermo, USA) and 250 μl of Opti-MEM culture medium (Gibco, USA). Tube B was made of KLF3-AS1, sh-KLF3-AS1, sh-Sirt1 vector or inhibitor-miR-138-5p, mimic-miR-138-5p sequences, and negative control (5 μl/well) in 250 μl of Opti-MEM culture medium. Tube A was incubated at room temperature for 5 min before mixed with tube B for 20 min. Then, cells were seeded into 6-well plates and incubated in an incubator for 6 h. Then, cells were continually cultured in refreshed DMEM culture medium (Gibco, USA) containing 10% FCS.

### CCK-8 assay

The transfected cells were seeded into 96-well plates. Three triplicates were set for each group. Cell viability was daily measured on the first 5 days. Then, 10 μl of CCK-8 solution (GLPBIO, USA) was added into each well for incubation at 37 °C for 2 h. The absorbance (OD) value at wavelength of 450 nm was measured using a microplate reader (Thermo, USA).

### ELISA

After cell transfection for 24 h, the concentrations of IL-1β and IL-18 in the supernatant of cardiomyocytes or serum of MI rat models were determined by ELISA kit (R&D, USA). The detection was strictly performed according to the instruction on ELISA kit.

### Dual-luciferase reporter assay

MiR-138-5p was predicted to bind with KLF3-AS1 by online database starBase (http://starbase.sysu.edu.cn/agoClipRNA.php?source=lncRNA). TargetScan software (http://www.targetscan.org/mamm_31/) predicted the binding site of miR-138-5p with Sirt1. Wide sequences and mutant sequences (mut-KLF3-AS1, wt-KLF3-AS1, mut-Sirt1 and wt-Sirt1) were designed and synthesized based on the predicted binding sites. Sequences were inserted into luciferase reporter vector (pGL3-Basic). Vectors were co-transfected with miR-138-5p mimic (30 nM) or miR-138-5p mimic negative controls (30 nM, GenePharma, Shanghai, China) into HEK293T cells (Shanghai SXBIO Biotech Co., Ltd). Cells were lysed with 100 μl of lysis buffer in a shaking bed at room temperature for 20 min. Cell suspension (50 μl) was incubated with 50 μl of luciferase solution (Promega, USA) before the intensity of Firefly luciferase was determined. Stop&Glo regent of 50 μl from Promega, USA, was fully mixed with cell suspension, and then, the intensity of Renilla luciferase was measured. The activity of Renilla luciferase was considered as internal control. The relative luciferase activity shall be the ratio of Firefly luciferase intensity and Renilla luciferase intensity. Triplicate wells were set for each group.

### Establishment of MI rat model

Animal studies were performed according to protocols and standards of laboratory animal studies. The design of animal models was approved by the local committee of Nanjing Lishui People’s Hospital. The MI rat models were established by ligation of the left anterior descending artery according to the Olivette method [26]. Rats were anesthetized by intraperitoneal injection of pentobarbital sodium (60 mg/kg) and operated with an incision in the chest. Anterior descending coronary artery was ligated with myocardial tissues. We also observed the color of the myocardium in the ligation zone blanched. The chest was closed, and the gas within the thoracic cavity was released. Rats all received post-operative care. Penicillin (200,000 U/day) was used for at least 3 days. Rats were kept separately until they are fully recovered. Rats in the sham group also received operation, but no ligation during the operation was performed. The left ventricular end-diastolic dimension (LVEDD) and left ventricular ejection fraction (LVEF) in both MI model group (*n* = 20) and sham group (*n* = 5) were monitored 1 week after operation to verify model establishment. MI rats were randomly grouped into four groups (*n* = 5) and correspondingly injected with equal volume (40 μg in 300 μl PBS/rat) of KLF3-AS1 exosomes (secreted by hMSCs transfected with KLF3-AS1 overexpression plasmid), NC exosomes (secreted by hMSCs transfected with KLF3-AS1 negative control), exosomes (secreted by hMSCs), or 300 μl of PBS for further experiments.

### TTC staining

Aorta of rats in each group was perfused with normal saline to wash away the blood before 1 ml of 0.3% Evans Blue staining solution was injected. Cardiac ventricle was transected into 2 mm of slices. The slices were then allowed to react in 1% triphenylterazolium chloride (TTC) at 37 °C for 15 min to determine the myocardial infarct zone. After staining, infarct areas blanched while non-infarct areas were in deep red.

### H&E staining

The myocardial tissues in the ischemic zone were fixed using 4% paraformaldehyde and embedded by paraffin. The tissues were sliced and stained with hematoxylin and eosin (H&E) for observation under a microscope.

### TUNEL assay

The apoptosis of cardiomyocyte in tissue slices and in H9c2 cells were determined by TUNEL assay. The apoptosis in pre-treated slices were determined using TUNEL fluorescence kit (In Situ Cell Death Detection Kit, TMR red, Roche, Indianapolis, IN, USA) according to instruction on the kits. Cell nucleus was stained with DAPI. Cell apoptosis was analyzed using image analyzer to calculate the positive cell rate.

### Statistical analysis

GraphPad 7.0 software was used for data analysis. All data were presented in the expression pattern of mean ± standard deviation. Statistical significance between two groups was evaluated with *T* test. Comparisons among multi-groups were determined using one-way analysis of variance with Dunnett’s multiple comparisons test for post hoc analysis. The Western blot, TTC staining, and TUNEL staining were analyzed using Image J software (version 1.6065; NIH). Significant difference was indicated by *P* < 0.05.

## Results

### Identification of hMSCs and exosomes derived from hMSCs

**Fig. 1:**
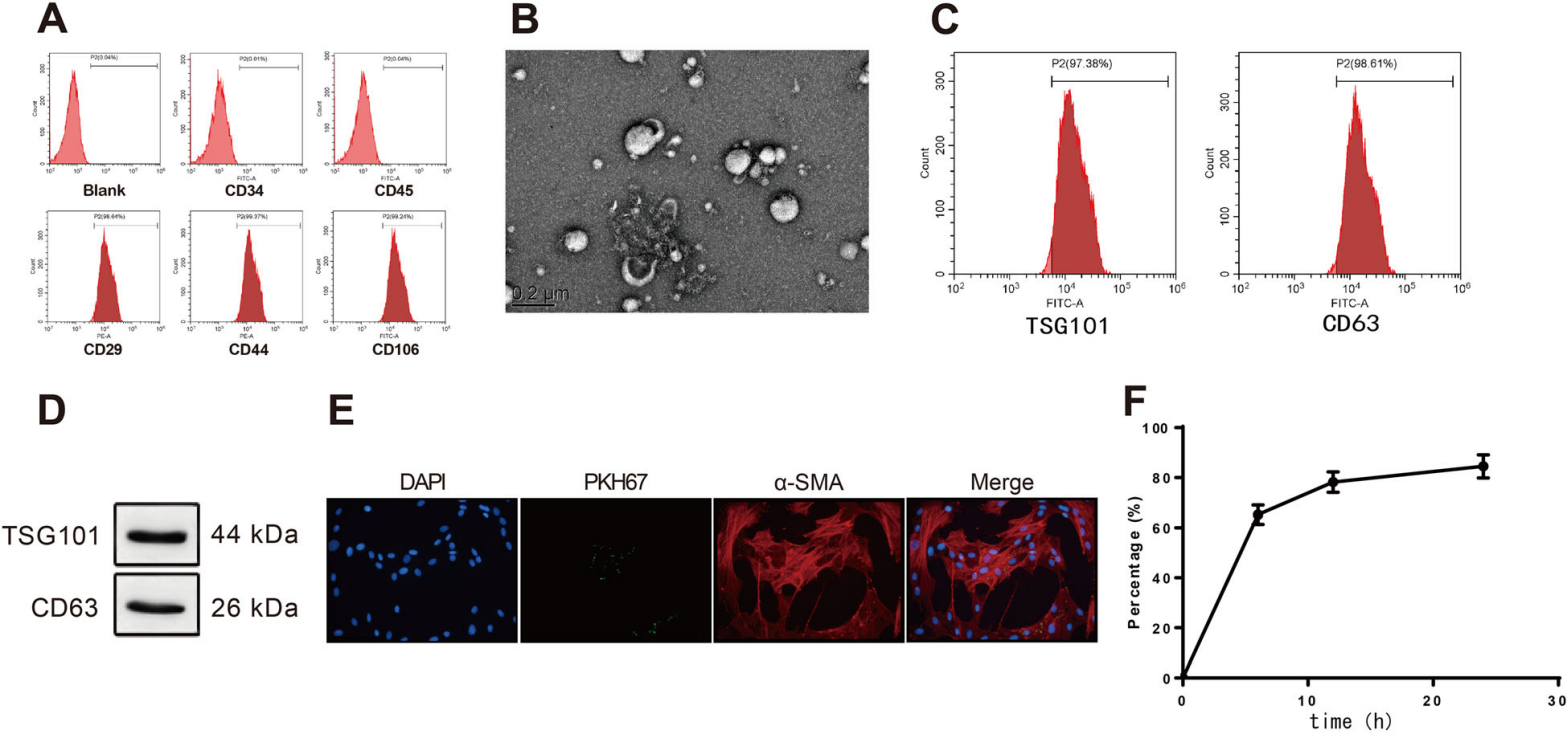
Identification of human mesenchymal stem cells and exosomes derived from human mesenchymal stem cells. Note: The biomarkers for HMSCs were measured including CD34, CD14, CD45, CD29, CD44, and CD106 (**a**). HMSCs, human mesenchymal stem cells. TEM demonstrated that the exosomes secreted by HMSCs were round vesicles (bars = 200 nm) (**b**). The detection on exosome-specific biomarker by FCM showed exosomes had positive expressions of TSG101 and CD63 (**c**). Western blot also detected the protein expressions of TSG101 and CD63 (**d**). Observation under a fluorescent microscope showed that PKH67-labeled exosomes were in green,α-SMA in red, and the cardiomyocyte nucleus DNA labeled by DAPI were in blue (× 400) (**e**). The ingestion of exosomes was measured by FCM (**f**). HMSCs, human mesenchymal stem cells; FCM, flow cytometry; TEM, transmission electron microscope

The hMSCs passaged for four times were in fusiform shape (Additional file 1: Figure S1A). After 3 days of incubation, hMSCs were grown into logarithmic growth phase, while after incubation for 6 and 7 days, cells were shifted into plateau phase and decline phase. Those observations suggested that the hMSCs used in this study were in logarithmic growth phase with stably morphologies and active cell viabilities (Additional file 1: Figure S1B). Data in previous study suggested that the positive biomarkers for hMSCs include CD29, CD44, CD13, CD34, CD146, and CD106, and negative biomarkers are CD34, CD14, CD45, CD11b, CD49d, and CD106 [27]. Evidence in previous study support CD106 as either a negative biomarker or a positive biomarker for hMSCs. In agreement with the previous study, in this study, we found positive expressions of CD29, CD44, and CD106 and negative expressions of CD34 and CD45 in hMSCs (Fig. 1a).

The diameter of exosomes ranges from 30 to 150 nm [28]. According to this standard, we randomly measured the diameters of 20 hMSC-derived exosomes and those exosomes have an average diameter of 63 nm (34 to 115 nm). The morphologies of exosomes under TEM are shown in Fig. 1b. The exosomes have complete structure and are round vesicles. FCM found positive expressions of exosomal biomarker TSG101 and CD63 in hMSC-secreted exosomes (Fig. 1c, d). After incubation with H9c2 cells for 12 h, PKH67 in exosomes was finally located on the cytoplasm of H9c2 cells (Fig. 1e). FCM showed the take-up of exosomes was increased with the incubation time. After cells were incubated with exosomes for 24 h, take-up of exosomes was saturated (Fig. 1f).

Furthermore, we measured the expression of KLF3-AS1 in exosomes derived from hMSCs. We detected KLF3-AS1 expression in hMSCs culture medium. RT-PCR also found that RNase could not alter the expression of KLF3-AS1 while RNase+TritonX-100 could suppress the expression of KLF3-AS1 in culture medium (Additional file 2: Figure S2A) (*P* < 0.001). Therefore, KLF3-AS1 in conditioned culture medium was membranous. To verify the stability of KLF3-AS1 expression, the expression of KLF3-AS1 in conditioned culture medium was separately determined at time point of 0 h, 4 h, 8 h, and 24 h at room temperature. The results indicated that KLF3-AS1 was stably expressed in conditioned culture medium after long-term preservation (Additional file 2: Figure S2B).

### KLF3-AS1 in exosome attenuates cell apoptosis and inflammatory response in MI rats

One week after surgery, 80% of rats (*n* = 32) survived (Additional file 3: Figure S3A). MI model group had increased LVEDD (Additional file 3: Figure S3B) and decreased LVEF (Additional file 3: Figure S3C) compared with that in the sham group (*P* < 0.001), indicating the successful establishment of MI rat model.

**Fig. 2:**
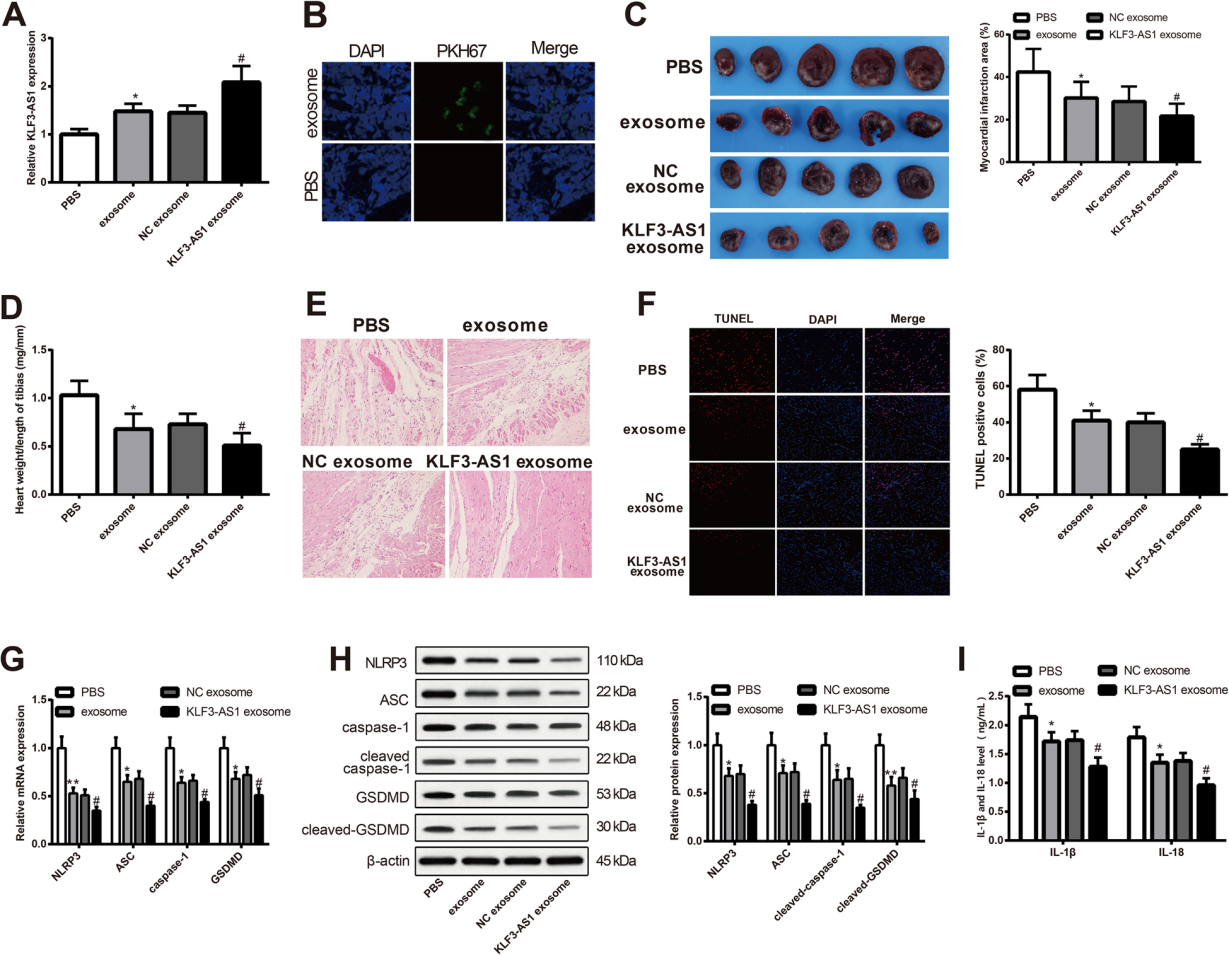
KLF3-AS1 in exosomes secreted by human mesenchymal stem cells can inhibit pyroptosis and attenuate MI in MI rat model. Note: After MI rats were injected with PBS, exosome, NC exosome, and KLF3-AS1 exosome, the expression of KLF3-AS1 was measured in each group (**a**). The PKH67-labeled exosomes were injected into MI rat model, and then, fluorescence microscope was used to clarify the location of PKH67 labeled exosomes in myocardial tissues (× 200) (**b**). TTC staining to observe the effect of KLF3-AS1 and exosomes on infarct zone of myocardial tissues (**c**). The heart weight/tibia length ratio of rats was calculated (**d**). H&E staining was performed to identify the morphology of myocardial tissues of MI rats (× 200) (**e**). Cell apoptosis was determined by TUNEL staining (× 200) (**f**). The mRNA and protein expressions of NLRP3, ASC, caspase-1/cleaved-caspase-1, and GSDMD/cleaved-GSDMD were measured by qRT-PCR (**g**) and Western blot (**h**). The inflammatory response in myocardial tissues was reflected by expressions of IL-1β and IL-18 by ELISA (**i**). *P < 0.05, **P < 0.01, ***P < 0.001, vs PBS group; ^#^*P* < 0.05, vs NC exosome group; MI, myocardial infarct; LVEDD, left ventricular end-diastolic dimension; LVEF, left ventricular ejection fraction

MI rat models were injected with KLF3-AS1 exosome, NC exosome, exosome, or PBS (*n* = 5) to verify the effect of exosomal KLF3-AS1 on MI rats. qRT-PCR demonstrated the expression of KLF3-AS1 in the exosome group, and the KLF3-AS1 exosome group was respectively higher than that in the PBS group and NC exosome group (Fig. 2a, *P* < 0.05). PKH67 green fluorescence was found in myocardium slices of rats with injection of exosomes, while no fluorescence was observed in rats injected with PBS (Fig. 2b). The fluorescence under fluorescence microscope demonstrated that exosomes were transferred to myocardium of MI rats.

Comparison on infarct zone showed the infarct zone in exosome and KLF3-AS1 exosome was smaller than those in the PBS group and NC exosome respectively (Fig. 2c, *P* < 0.05). Exosome group had decreased heart weight/tibia length ratio compared with the PBS group, while the KLF3-AS1 exosome group had decreased heart weight/tibia length ratio in comparison with that in the NC exosome group (Fig. 2d). Myocardium tissues in each group were stained with H&E (Fig. 2e). Staining on PBS group showed that the cardiomyocytes were swelling and cell nucleus arranged unregularly, in addition to enlarged mesenchyme of cells and infiltration of neutrophile granulocyte. The condition of cardiomyocytes was attenuated in both exosome and NC exosome group. Rats in the KLF3-AS1 exosome group had the least myocardial injury than the rest three groups.

Cell apoptosis rate and inflammatory response in exosome group and KLF3-AS1 exosome were ameliorated when respectively compared with the PBS group and NC exosome group (Fig. 2f, *P* < 0.05). The expressions of NLRP3, ASC, and caspase-1 in the exosome group and KLF3-AS1 exosome group were lower than those in the PBS and NC exosome group, respectively (Fig. 2g, h, *P* < 0.05). Measurement on inflammatory cytokines showed the pro-inflammatory cytokines IL-1β and IL-18 were suppressed in the exosome group and KLF3-AS1 exosome group (Fig. 2i, *P* < 0.05). The detection of myocardial injury, infarct zone, apoptosis rate, and inflammation of MI rat model supported that KLF3-AS1 in hMSC-derived exosomes can inhibit cell pyroptosis and attenuate MI in MI rat model.

### Exosomes derived from hMSCs affect pyroptosis of cardiomyocytes

**Fig. 3:**
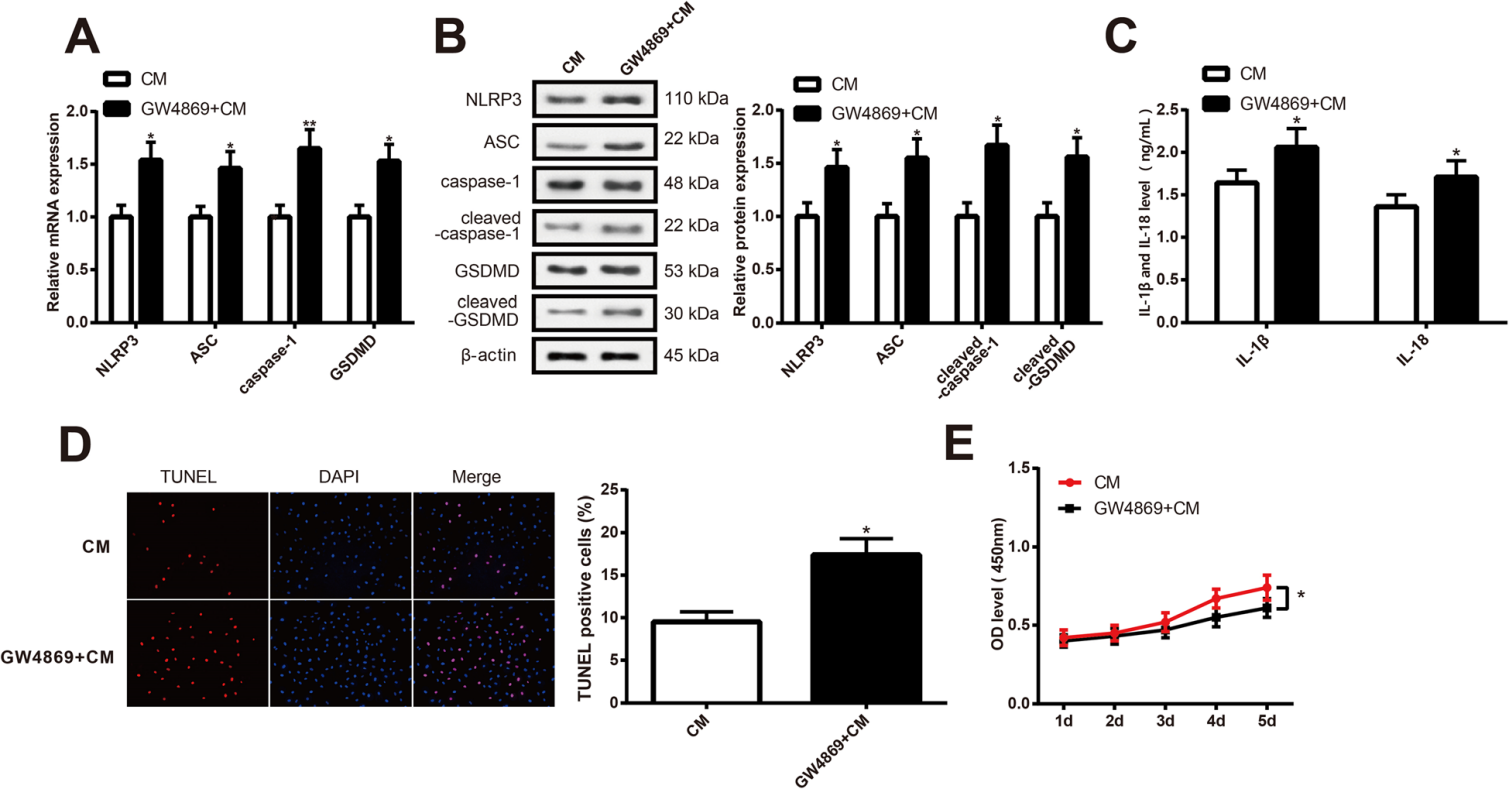
Exosome inhibitor can reverse the effect of hMSC-conditioned culture medium on pyroptosis of cardiomyocytes. Note: mRNA and protein expressions of NLRP3, ASC, caspase-1/cleaved-caspase-1, and GSDMD/cleaved-GSDMD in hypoxia cardiomyocytes were determined by qRT-PCR (**a**) and Western blot (**b**). Concentration of IL-1β and IL-18 in culture medium of hypoxia cardiomyocytes was measured by ELISA (**c**). Cell apoptosis was determined by TUNEL staining (× 200) (**d**). Cell viability of hypoxia cardiomyocytes was determined by CCK-8 (**e**). *P < 0.05, **P < 0.01, vs CM group; CM, conditioned culture medium

Exosome inhibitor (GW4869) was used to inhibit the function of exosomes. The conditioned culture medium of hMSCs was used to culture cardiomyocytes. The detection of pyroptosis-related factors showed that the expression levels of NLRP3, ASC, cleaved-caspase-1, and cleaved-GSDMD were elevated in the GW4869+CM group when compared with the CM group (Fig. 3a, b, *P* < 0.05). Furthermore, the GW4869+CM group had increased expression levels of IL-1β and IL-18, enhanced apoptosis rate, and suppressed cell viability than those in the CM group (Fig. 3c–e, *P* < 0.05). Collectively, exosome inhibitor can reverse the effect of hMSC-conditioned culture medium on pyroptosis of cardiomyocytes.

### KLF3-AS1 in exosome attenuates pyroptosis of cardiomyocytes

**Fig. 4:**
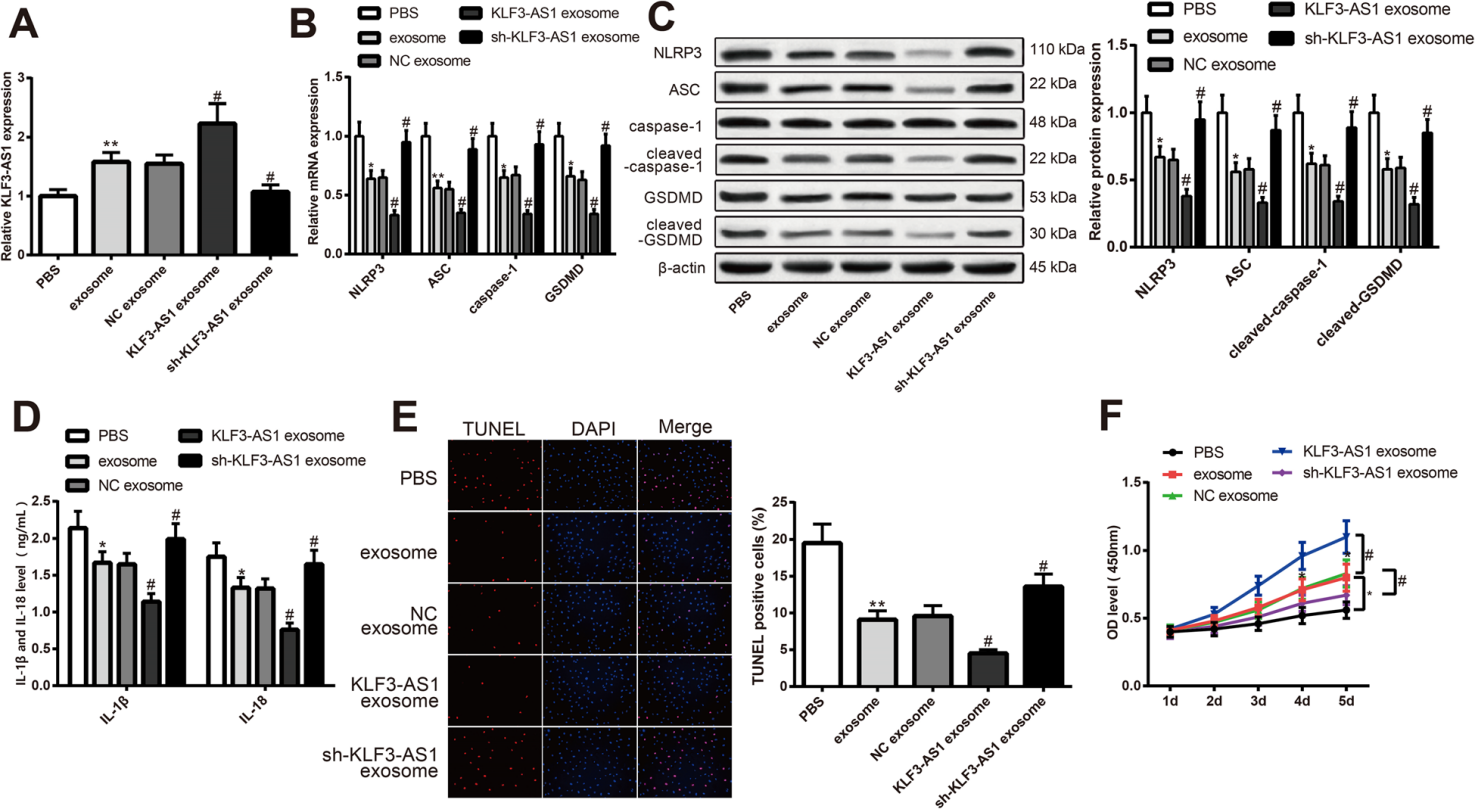
KLF3-AS1 in exosomes secreted by human mesenchymal stem cells can inhibit pyroptosis of hypoxia cardiomyocytes. Note: The secreted exosomes were incubated with hypoxia cardiomyocytes and qRT-PCR was applied to measure expression of KLF3-AS1 in cardiomyocytes (**a**). mRNA and protein expressions of NLRP3, ASC, caspase-1/cleaved-caspase-1 and GSDMD/cleaved-GSDMD in hypoxia cardiomyocytes were determined by qRT-PCR (**b**) and Western blot (**c**). Concentration of IL-1β and IL-18 in culture medium of hypoxia cardiomyocytes was measured by ELISA (**d**). Cell apoptosis rate of cardiomyocytes was detected by TUNEL staining (× 200) (**e**). Viability of cardiomyocytes was measured by CCK-8 assay (**f**). * *P* < 0.05, **P < 0.01, vs PBS group; ^#^ *P* < 0.05, vs NC exosome group; qRT-PCR, qRT-PCR, quantitative reverse transcription-polymerase chain reaction

To explore the effect of KLF3-AS1 on pyroptosis of cardiomyocytes, exosomes in each group were separately incubated with hypoxia cardiomyocytes. PBS incubation was set as control. The expression of KLF3-AS1 in each group was measured. The expression of KLF3-AS1 in the exosome group and in the KLF3-AS1 exosome group was higher than that in the PBS group and the NC exosome group, respectively. Nevertheless, the expression of KLF3-AS1 in the sh-KLF3-AS1 exosome group was lower than that in the NC exosome group (Fig. 4a, *P* < 0.05). As shown in Fig. 4b, c, the exosome group and KLF3-AS1 exosome group had decreased expressions of NLRP3, ASC, cleaved-caspase-1, and cleaved-GSDMD compared with that in the PBS group and NC exosome group, respectively (*P* < 0.05). The sh-KLF3-AS1 exosome group had elevated expressions of NLRP3, ASC, cleaved-caspase-1, and cleaved-GSDMD when compared with the NC exosome group (Fig. 4b, c, *P* < 0.05).

Inflammatory response in the exosome group and KLF3-AS1 exosome group was attenuated than those in the PBS and NC exosome group, evidenced by decreased expressions of IL-1β and IL-18 (Fig. 4d, *P* < 0.05). Increased expressions of IL-1β and IL-18 were found in the sh-KLF3-AS1 group in comparison with that in the NC exosome group (Fig. 4d, *P* < 0.05). In comparison with the PBS group and NC exosome group, cell apoptosis rate in the exosome group and KLF3-AS1 exosome was suppressed (Fig 4e, *P* < 0.05). The apoptosis rate in the sh-KLF3-AS1 group was evidently higher than that in the NC exosome group (Fig. 4e, *P* < 0.05). Detection on cell viability showed that the exosome group and KLF3-AS1 exosome group had increased cell viability than those in the PBS group and NC exosome group (Fig. 4f–h, *P* < 0.05). The cell viability in the sh-KLF3-AS1 group suppressed when compared with the NC exosome group (Fig. 4f–h, *P* < 0.05). Collectively, KLF3-AS1 in hMSC-derived exosomes can hamper the pyroptosis of hypoxia cardiomyocytes.

### KLF3-AS1 act as a ceRNA to react with miR-138-5p to regulate Sirt1

**Fig. 5:**
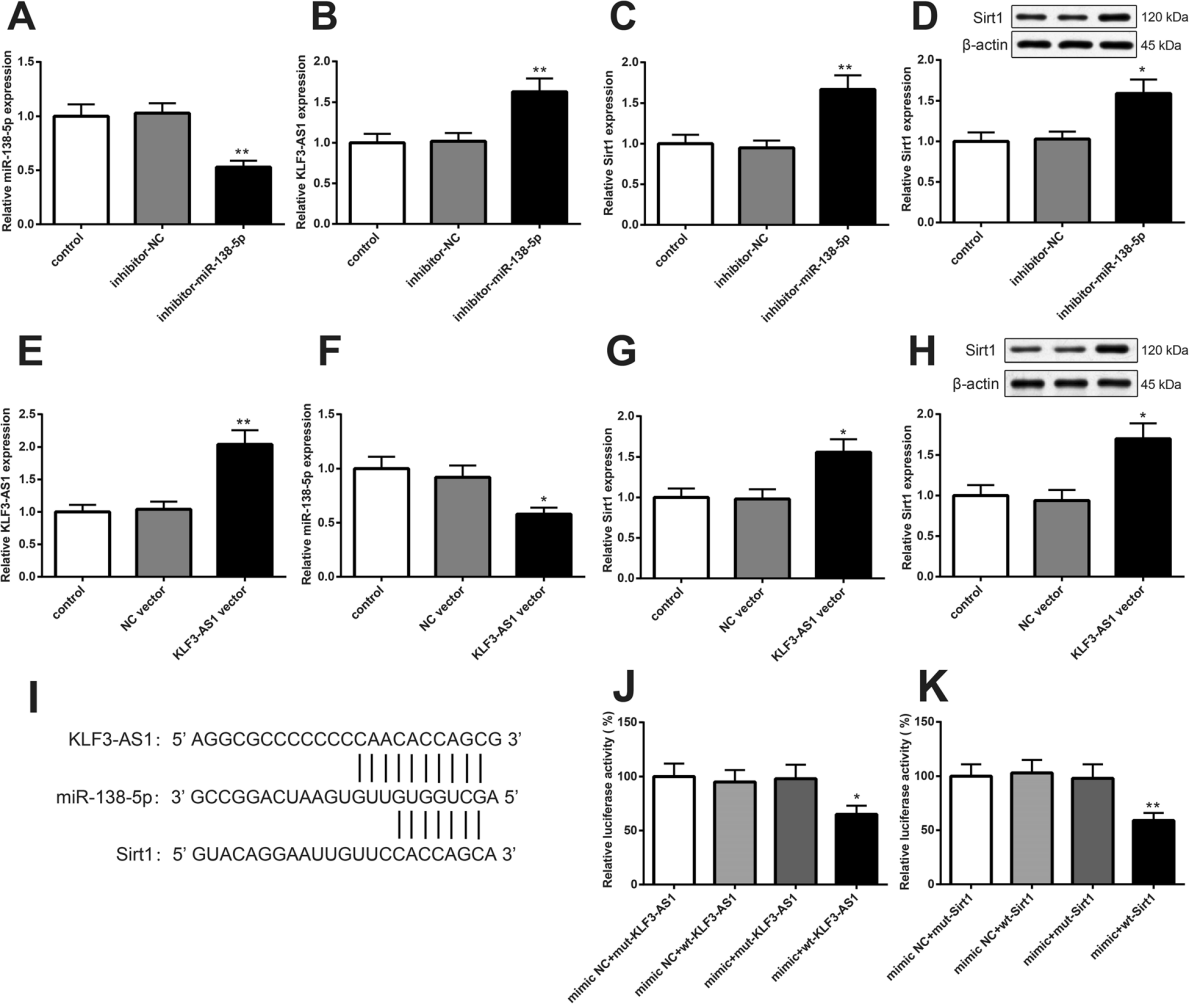
KLF3-AS1 can bind with miR-138-5p to regulate expression of Sirt1. Note: After cardiomyocytes were transfected with miR-138-5p inhibitor, the expressions of miR-138-5p (**a**), KLF3-AS1 (**b**), and Sirt1 (**c**, **d**) were determined by qRT-PCR and Western blot. Then, cardiomyocytes transfected with KLF3-AS1 overexpression vector were measured for expressions of miR-138-5p (**f**), KLF3-AS1 (**e**), and Sirt1 (**g**, **h**). The predicted bind sites of miR-138-5p with KLF3-AS1 and Sirt1 are listed in **i**. Dual dual-luciferase reporter assay confirmed the binding of miR-138-5p with KLF3-AS1 (**j**) and with Sirt1 (**k**). *P < 0.05, **P < 0.01, vs control group or mimic+mut-KLF3-AS1 group or mimic+mut-Sirt1 group; qRT-PCR, quantitative reverse transcription-polymerase chain reaction

After cardiomyocytes were transfected with miR-138-5p inhibitor, the expression of miR-138-5p was suppressed (Fig. 5a, *P* < 0.05), while the expressions of KLF3-AS1 and Sirt1 were upregulated (Fig. 5b–d, *P* < 0.05). Transfection of KLF3-AS1 overexpression vector in cardiomyocytes results in the upregulation of KLF3-AS1 (Fig. 5e, *P* < 0.05) and Sirt1 (Fig. 5g, h, *P* < 0.05) and the downregulation of miR-138-5p (Fig. 5f, *P* < 0.05). The predicted bind sites of miR-138-5p with KLF3-AS1 and the bind sites of miR-138-5p with Sirt1 are displayed in Fig. 5i. Dual-luciferase reporter assay demonstrated that the luciferase activity of mimic+wt-KLF3-AS1 group and mimic+wt-Sirt1 was suppressed compared with that in mimic+mut-KLF3-AS1 group and mimic+mut-Sirt1 group, respectively (Fig. 5j, k, *P* < 0.05). KLF3-AS1 can bind with miR-138-5p, and miR-138-5p can target Sirt1.

### KLF3-AS1 in exosomes inhibits pyroptosis of cardiomyocytes through downregulating miR-138-5p

**Fig. 6:**
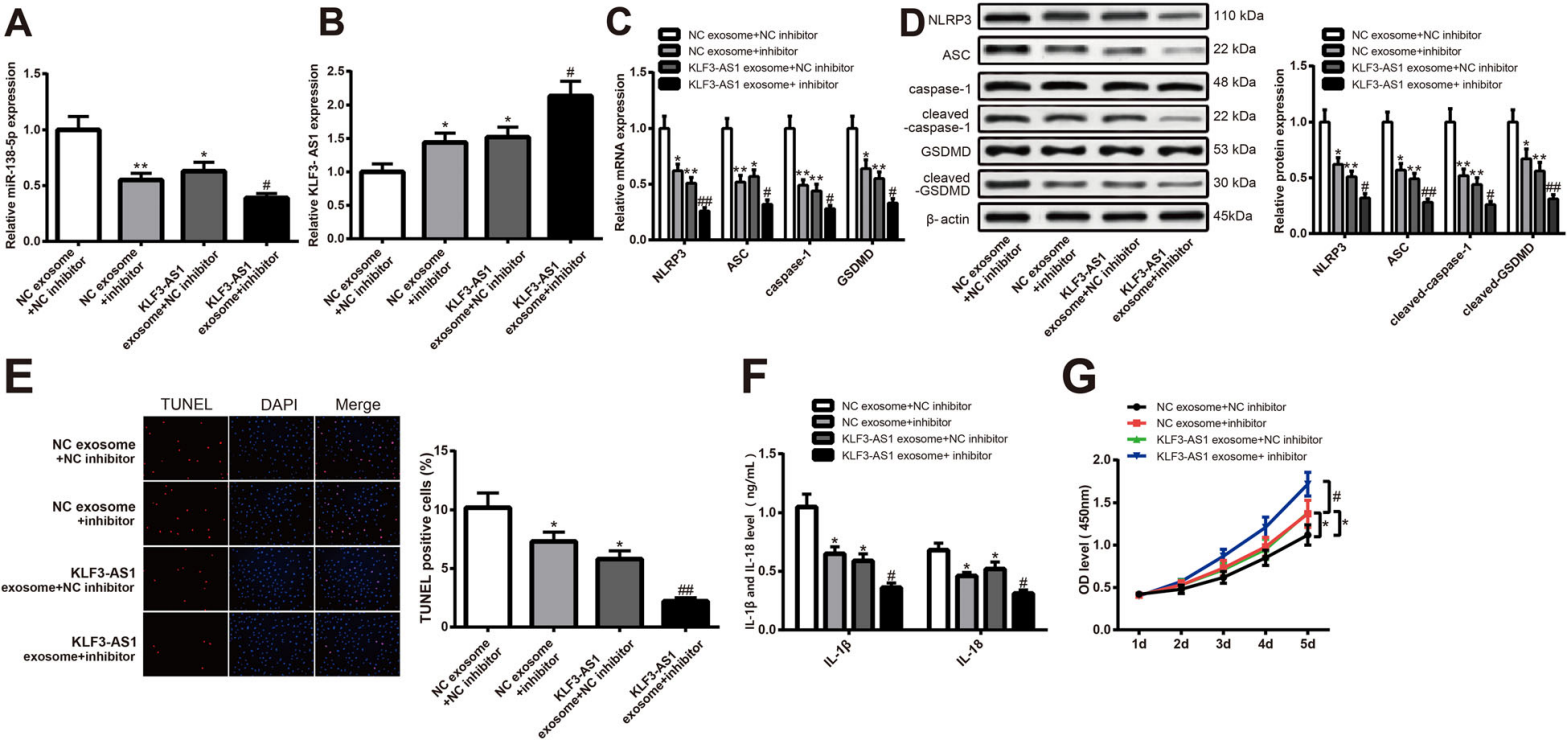
KLF3-AS1 secreted by human mesenchymal stem cells hampers pyroptosis of cardiomyocytes by downregulating miR-138-5p. Note: After cells were transfected with miR-138-5p inhibitor or incubated with KLF3-AS1 exosomes, the expressions of miR-138-5p and KLF3-AS1 were measured by qRT-PCR (**a**, **b**). The expressions of NLRP3, ASC, caspase-1/cleaved-caspase-1, and GSDMD/cleaved-GSDMD in hypoxia cardiomyocytes were determined by both qRT-PCR (**c**) and Western blot (**d**). Cell apoptosis was determined by TUNEL staining, and DAPI was used to stain cell nucleus (× 200) (**e**). Inflammatory response was measured by detecting expressions of IL-1β and IL-18 using ELISA (**f**). Cell viability was determined by CCK-8 on cell viability (**g**). *P < 0.05, **P < 0.01, vs NC exosome + NC inhibitor group; ^#^*P* < 0.05, ^##^*P* < 0.05, vs NC exosome + inhibitor group; qRT-PCR, quantitative reverse transcription-polymerase chain reaction

Transfection of miR-138-5p inhibitor in cardiomyocytes results in the downregulation of miR-138-5p and upregulation of KLF3-AS1. Then, the hypoxia cardiomyocytes were incubated with KLF3-AS1 exosome/NC exosome, and the results showed the expression of miR-138-5p was further suppressed and KLF3-AS1 was upregulated (KLF3-AS1 exosome + inhibitor vs NC exosome + inhibitor, Fig. 6a, b, *P* < 0.05). Transfection of miR-138-5p inhibitor or/and incubation with KLF3-AS1 exosome in hypoxia cardiomyocytes can lead to downregulated expressions of NLRP3, ASC, cleaved-caspase-1, cleaved-GSDMD (Fig. 6c, d), IL-1β, and IL-18 (Fig. 6f, *P* < 0.05); inhibited cell apoptosis rate (Fig. 6e *P* < 0.05); and upregulated cell viability (Fig. 6g, *P* < 0.05). Those results showed that transfection of miR-138-5p inhibitor and upregulated expression of KLF3-AS1 could hamper pyroptosis of cardiomyocytes. miR-138-5p inhibitor could enhance the inhibitory effect of hMSC-derived exosomes on pyroptosis of cardiomyocytes. hMSC-derived exosomes hamper pyroptosis of cardiomyocytes by downregulating the expressions of miR-138-5p.

### KLF3-AS1 in exosomes inhibits pyroptosis of cardiomyocytes through upregulating Sirt1

**Fig. 7:**
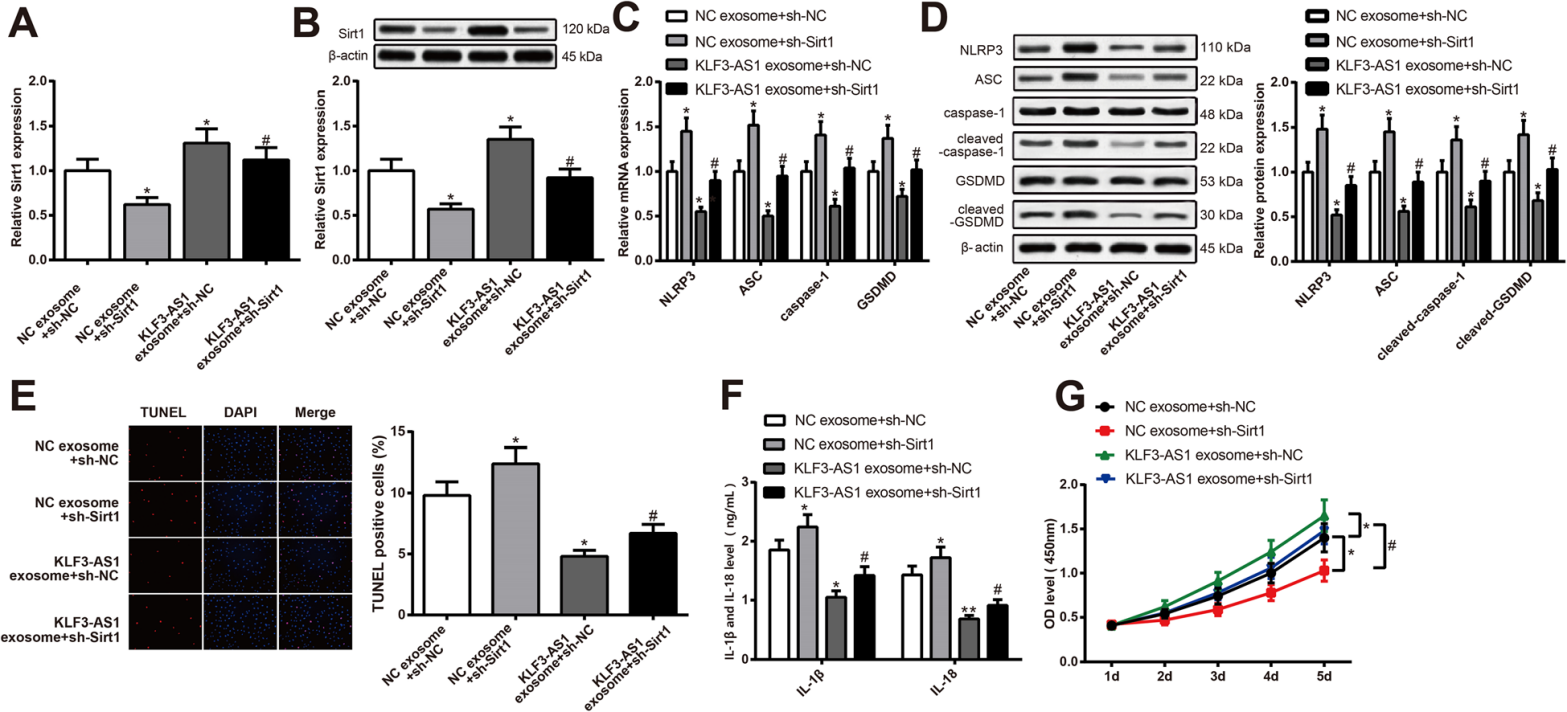
KLF3-AS1 secreted by human mesenchymal stem cells hampers pyroptosis of cardiomyocytes by upregulating Sirt1. Note: Hypoxia cardiomyocytes were treated with sh-sirt1 or/and KLF3-AS1 exosome. qRT-PCR (**a**) and Western blot (**b**) showed that KLF3-AS1 exosome could upregulate while sh-sirt1 could downregulate expression of Sirt1. The effect of Sirt1 knock down and KLF3-AS1 exosome on mRNA and protein expressions of NLRP3, ASC, caspase-1/cleaved-caspase-1, and GSDMD/cleaved-GSDMD was determined by qRT-PCR (**c**) and Western blot (**d**). Cell apoptosis of hypoxia cardiomyocytes was determined by TUNEL staining (× 200) (**e**). Concentration of IL-1β and IL-18 was determined by ELISA (**f**). Cell viability was determined by CCK-8 (**g**). *P < 0.05, **P < 0.01, vs NC exosome + sh-NC group; ^#^*P* < 0.05, ^##^*P* < 0.01, vs NC exosome + sh-Sirt1 group; qRT-PCR, quantitative reverse transcription-polymerase chain reaction

Knock-down of Sirt1 expression by sh-Sirt1 (Fig. 7a, b) could upregulate expressions of NLRP3, ASC, cleaved-caspase-1, cleaved-GSDMD (Fig. 7c, d), IL-1β, and IL-18 (Fig. 7f); increase cell apoptosis rate (Fig. 7e); and inhibit cell viability (Fig. 7g), therefore aggravating cell pyroptosis of cardiomyocytes (NC exosome + sh-Sirt1 vs NC exosome + sh-NC, *P* < 0.05). KLF3-AS1 exosome treatment could reverse the unfavorable effect of sh-Sirt1 on cardiomyocytes, thus attenuating the pyroptosis of cardiomyocytes. Downregulation of Sirt1 could reverse the inhibitory effect of hMSC-derived exosomes on cell pyroptosis of hypoxia cardiomyocytes. KLF3-AS1 in exosomes can upregulate the expression of Sirt1 to hamper cell pyroptosis. Collectively, KLF3-AS1 in hMSC-derived exosomes can inhibit cell pyroptosis of hypoxia cardiomyocytes and ameliorate MI through miR-138-5P/Sirt1 axis.

## Discussion

To determine the treatment strategies of MI necessitates the understanding on pathology of pyroptosis in MI at both cellular and molecular levels. Cell pyroptosis is regarded as programmed cell death mediated by caspase-1 to trigger inflammatory responses in response to pathogen and endogenous mediators [29]. Our study demonstrated that exosomal lncRNA KLF3-AS1 derived from hMSCs can inhibit cell viability, inflammatory response, and cell pyroptosis of cardiomyocytes, and therefore attenuate MI progression through regulating miR-138-5p/Sirt1 axis.

Extracellular vesicles were isolated from hMSCs whose diameters were measured. The observation under TEM and diameter measurement confirmed that the extracellular vesicles derived from hMSCs are exosomes. Then, to ensure the stability of KLF3-AS1 in exosomes, we used RNase and TritonX-100 to treat the conditioned culture medium and then measured the expression of KLF3-AS1 in culture medium of hMSCs to ensure KLF3-AS1 was membranous. Those results provided solid ground for our further experiments.

Detection on pyroptosis-related cytokines and H&E staining on rat MI models and hypoxia cardiomyocytes supported that KLF3-AS1 in exosomes secreted by hMSCs can suppress the expression levels of NLRP3, ASC, caspase-1, GSDMD, IL-1β, and IL-18 in addition to improving cell morphologies of hypoxia cardiomyocytes. Different from apoptosis, during pyroptosis, the integrity of cell membrane was breached, leading to inflammatory response activated by releasing of intracellular contents [30]. The complexes of cytosolic compartment could accumulate to form inflammasomes which initiates sensors of NLR family members and inflammatory caspase, consequently leading to recruitment of ASC and caspase-1 which was necessary for the cleavage of GSDMD pro-IL-1β and pro-IL-18 [31]. Therefore, in this case, pyroptosis was initiated and matured expressions of IL-1β and IL-18 were secreted [32]. The observation in this study found assembly of NLRP3 inflammasome, activation form of caspase-1 along with release of IL-1β and IL-18 in exosome-treated hypoxia cells, supporting the implication of KLF3-AS1 or exosomes secreted by hMSCs in pyroptosis of hypoxia cardiomyocytes. In this study, hypoxia cell models were established to simulate myocardial ischemia and infarction in vivo. Therefore, the result of this study in MI rat models and hypoxia cell models may imply that KLF3-AS1 in hMSC-derived exosomes can suppress the pyroptosis of cardiomyocytes after MI. Understanding the pathology of pyroptosis helps us to identify the involvement of KLF3-AS1 herein. Pyroptosis is characterized by intensive inflammatory response, in which caspase-1 have a vital role to play, as release of inflammatory cytokines IL-1β and IL-18 was triggered by active form of caspase-1 [33]. Therefore, we hypothesized KLF3-AS1 may regulate caspase-1 directly or indirectly during pyroptosis and MI.

In order to explore the mechanism herein, we applied dual-luciferase reporter assay and found KLF3-AS1 could serve as a ceRNA to sponge miR-138-5p and thus mediate Sirt1. In osteoarthritis, KLF3-AS1 derived from hMSCs can facilitate chondrocyte by acting as a ceRNA to bind with miR-206 and regulate the expression of GIT1 [22]. Consistent with the above results, our results demonstrated that cardiomyocytes with transfection of miR-138-5p inhibitor had upregulated expression of KLF3-AS1 and Sirt1. Sirt1 is found as an inflammation regulator in cardiovascular diseases and participates in immune responses [34, 35]. Sirt1 plays a central role in regulating a variety of cellular processes related to heart development and cardiovascular diseases [36, 37]. Sirt1 is found to be downregulated in MI, and overexpression of Sirt1 can effectively ameliorate the myocardial injury induced by MI [38]. Data on vascular endothelial cells clarified that Sirt1 can suppress the expressions of NLRP3 and ASC in addition to inhibition on the maturation of caspase-1 and inflammatory cytokine IL-1β, indicating the suppression role of Sirt1 on the activation of NLRP3 inflammasome and the protective role of Sirt1 on vascular endothelial cells [39]. Among all the inflammasomes, NLRP3 inflammasome is the most important one belonging to the effector of the innate immune system which can facilitate the maturation and release of IL-1β and IL-18 cytokines, consequently initiating inflammatory responses [40]. The implication of NLRP3 inflammasome was reported in several diseases, including ischemia reperfusion injury on the brain, heart, kidney, and testis; neurodegenerative disease; and cerebral tumors as well [41]. The ischemia would aggravate the proliferation viability of cardiomyocytes and damage the heart contractibility [42], unfavorable for the maintenance of heart function. Results in the current study supported that KLF3-AS1 derived from hMSCs can upregulate the expressions of NLRP3 inflammasome-related factors and inhibit cell pyroptosis of cardiomyocytes and attenuate MI. Therefore, it is reasonable to speculate that KLF3-AS1 secreted from hMSCs by acting as a ceRNA to sponge miR-138-5p facilitates the expression of Sirt1 to ameliorate inflammatory response, pyroptosis, and MI. The inhibition of NLRP3 inflammasome by Sirt1 was involved in multiple diseases. For instance, the suppression of salidroside in activation of NLRP3 inflammasome was associated with the role of Sirt1 [43]. Moreover, the implication of SIRT1/NOX4/ROS pathway was uncovered in anti-pyroptosis role of liraglutide against TNF-α and hypoxia-stimulated cardiomyoblasts [44]. Interestingly, Sirt1 is cleaved by caspase-1 which is activated by NLRP3 inflammasome, and after the activation of NLRP3 inflammasome, the cleavage of Sirt1 by caspase-1 could reduce the function of Sirt1 in cells [45]. Taken together, Sirt1 can inhibit the activation of NLRP3 inflammasome, and thus attenuate pyroptosis and MI.

## Conclusion

In summary, data in this study supported that exosomes secreted by hMSCs can increase the expression of KLF3-AS1. Experiments on cardiomyocytes and on rats injected with exosomes which were transfected with overexpression of KLF3-AS1 indicated that KLF3-AS1 in hMSC-secreted exosomes could attenuate pyroptosis of cardiomyocytes in rat models and of hypoxic cardiomyocytes. KLF3-AS1 by serving as a ceRNA to sponge miR-138-5p can mediate the expression of Sirt1, so as to regulate pyroptosis of cardiomyocytes and MI progression. Investigation on inflammatory caspases and pyroptosis in MI is required to advance our understanding to prevent and treat MI in human patients.

## Supplementary information

**Additional file 1: Figure S1.** Identification of HMSCs. Note: The morphology of the 4th generations of HMSCs was observed under a microscope (× 40) (A). Cell growth of HMSCs was monitored, indicating for typical curve for exponential growth (B). HMSCs, human mesenchymal stem cells.

**Additional file 2: Figure S2.** The expression of KLF3-AS1 in exosomes derived from HMSCs. Note: RNase and RNase+TritonX-100 were added in the culture medium for HMSCs before KLF3-AS1 expression in exosomes derived from HMSCs was determined by qRT-PCR (A). Relative expressions of KLF3-AS1 in exosomes derived from HMSCs were measured after culture medium was maintained at room temperature for 0 h, 4 h, 8 h or 24 h (B). HMSCs, human mesenchymal stem cells.

**Additional file 3: Figure S3.** Identification of MI rat model. Note: The survival rate of MI rat models in post-operational one week (A). The LVEDD (B) and LVEF (C) of rats in sham group and MI model group in post-operational one week. * *P* < 0.05, *** *P* < 0.001, vs Sham group; MI, myocardial infarct; LVEDD, left ventricular end-diastolic dimension; LVEF, LVEF, left ventricular ejection fraction.

## Data Availability

The datasets used and/or analyzed during the current study are available from the corresponding author on reasonable request.

## Abbreviations

ceRNAs: Competing endogenous RNAs
DAPI: 4′, 6-diamidino-2-phenylindole
FCM: Flow cytometry
FCS: Fetal calf serum
H&E: Hematoxylin and eosin
hMSCs: Human mesenchymal stem cells
lncRNAs: Long non-coding RNAs
LVEDD: Left ventricular end-diastolic dimension
LVEF: Left ventricular ejection fraction
MI: Myocardial infarction
miRNAs: microRNAs
MSCs: Mesenchymal stem cells
TEM: Transmission electron microscope
TTC: Triphenylterazolium chloride
