# Supplementary figures and images for: LncRNA KLF3-AS1 in human mesenchymal stem cell-derived exosomes ameliorates pyroptosis of cardiomyocytes and myocardial infarction through miR-138-5p/Sirt1 axis
Source: Stem Cell Res Ther. 2019 Dec 17;10:393. doi: 10.1186/s13287-019-1522-4 (PMC6918658; doi:10.1186/s13287-019-1522-4)

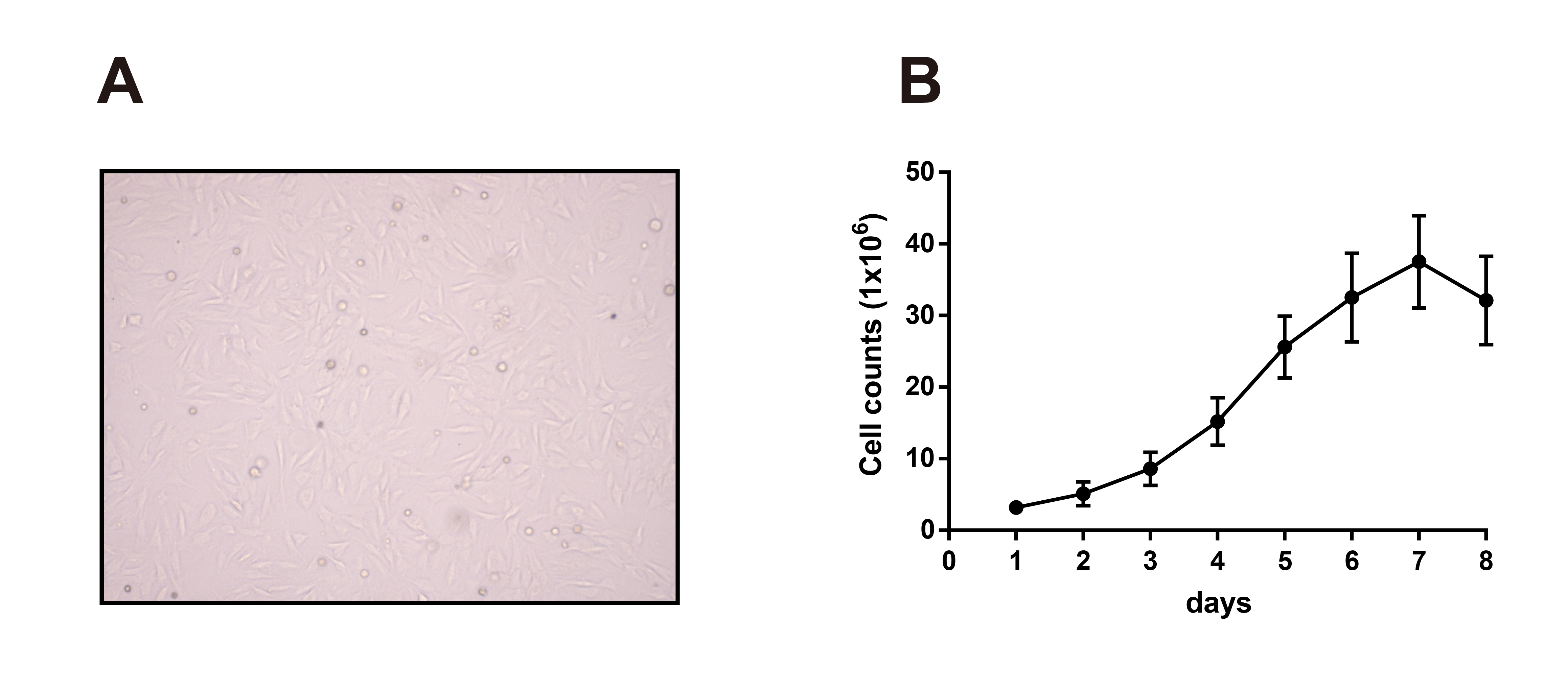

Supplement: Supplementary file 1 — Additional file 1: Figure S1. Identification of HMSCs. Note: The morphology of the 4th generations of HMSCs was observed under a microscope (× 40) (A). Cell growth of HMSCs was monitored, indicating for typical curve for exponential growth (B). HMSCs, human mesenchymal stem cells. [file 13287_2019_1522_MOESM1_ESM.jpg]

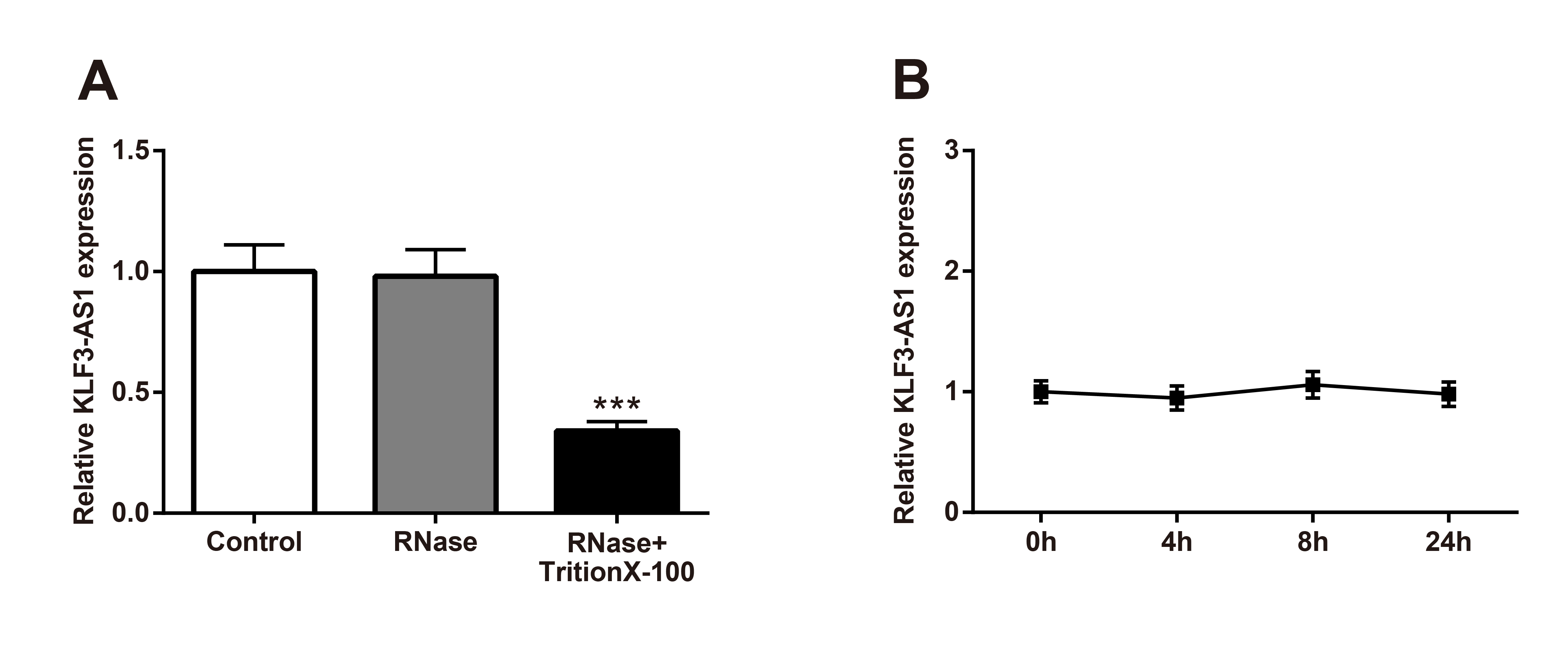

Supplement: Supplementary file 2 — Additional file 2: Figure S2. The expression of KLF3-AS1 in exosomes derived from HMSCs. Note: RNase and RNase+TritonX-100 were added in the culture medium for HMSCs before KLF3-AS1 expression in exosomes derived from HMSCs was determined by qRT-PCR (A). Relative expressions of KLF3-AS1 in exosomes derived from HMSCs were measured after culture medium was maintained at room temperature for 0 h, 4 h, 8 h or 24 h (B). HMSCs, human mesenchymal stem cells. [file 13287_2019_1522_MOESM2_ESM.jpg]

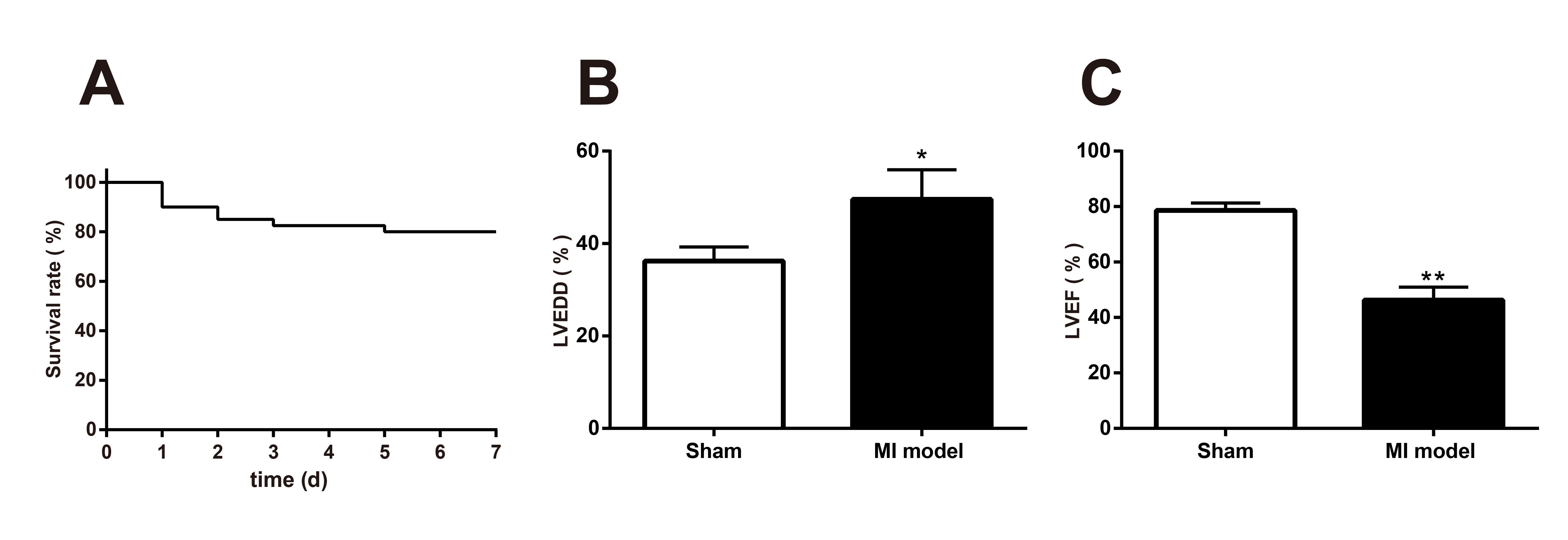

Supplement: Supplementary file 3 — Additional file 3: Figure S3. Identification of MI rat model. Note: The survival rate of MI rat models in post-operational one week (A). The LVEDD (B) and LVEF (C) of rats in sham group and MI model group in post-operational one week. * P < 0.05, *** P < 0.001, vs Sham group; MI, myocardial infarct; LVEDD, left ventricular end-diastolic dimension; LVEF, LVEF, left ventricular ejection fraction. [file 13287_2019_1522_MOESM3_ESM.jpg]
